# Feasibility study on atrial fibrillation ablation with pulsed field ablation and concomitant occlusion of the left atrial appendage

**DOI:** 10.1093/europace/euae176

**Published:** 2024-06-25

**Authors:** Jennifer Beney, Roberto Galea, Georgios Siontis, Christoph Gräni, Thomas Kueffer, Nicolas Brugger, Tobias Reichlin, Lorenz Räber, Laurent Roten

**Affiliations:** Department of Cardiology, Inselspital, Bern University Hospital, University of Bern, Freiburgstrasse, CH-3010 Bern, Switzerland; Department of Cardiology, Inselspital, Bern University Hospital, University of Bern, Freiburgstrasse, CH-3010 Bern, Switzerland; Department of Cardiology, Inselspital, Bern University Hospital, University of Bern, Freiburgstrasse, CH-3010 Bern, Switzerland; Department of Cardiology, Inselspital, Bern University Hospital, University of Bern, Freiburgstrasse, CH-3010 Bern, Switzerland; Department of Cardiology, Inselspital, Bern University Hospital, University of Bern, Freiburgstrasse, CH-3010 Bern, Switzerland; Department of Cardiology, Inselspital, Bern University Hospital, University of Bern, Freiburgstrasse, CH-3010 Bern, Switzerland; Department of Cardiology, Inselspital, Bern University Hospital, University of Bern, Freiburgstrasse, CH-3010 Bern, Switzerland; Department of Cardiology, Inselspital, Bern University Hospital, University of Bern, Freiburgstrasse, CH-3010 Bern, Switzerland; Department of Cardiology, Inselspital, Bern University Hospital, University of Bern, Freiburgstrasse, CH-3010 Bern, Switzerland

**Keywords:** Atrial fibrillation, Catheter ablation, Left atrial appendage occlusion, Pulmonary vein isolation, Pulsed field ablation

## Abstract

**Aims:**

Atrial fibrillation (AF) ablation and left atrial appendage occlusion (LAAO) are increasingly performed as individual procedures. Pulsed field ablation (PFA) has significantly reduced procedure duration and may be advantageous for the combined approach.

**Methods and results:**

We have launched a programme for simultaneous AF ablation using PFA and LAAO for patients qualifying for both treatments and excluding those with a complex anatomy. We compare procedure duration and fluoroscopy time against individual procedures (either AF ablation or LAAO alone), all performed by the same operators and using consistent technologies. We performed the combined procedure in 10 patients (50% males; median age 70 years) and excluded 2 patients (17%) because of a complex left atrial appendage anatomy. No death, stroke, or major bleeding events, including pericardial effusion, occurred. For single-procedure comparison, 207 AF ablation procedures and 61 LAAO procedures were available. The total median procedure duration was 79 min (range 60–125) for the combined procedure, 71 min (25–241) for individual AF ablation (51 min without and 78 min with 3-dimensional electroanatomic mapping), and 47 min (15–162) for individual LAAO. The respective fluoroscopy times were 21 (15–26), 15 (5–44), and 10 (3–50) min. For the combined procedure, femoral vein access to last PFA application lasted 49 min (34–93) and LAAO added 20 min (15–37).

**Conclusion:**

Simultaneous PFA-based AF ablation and LAAO in carefully selected patients is feasible and safe and can be executed within a short overall procedure duration.

## Graphical Abstract

**Graphical Abstract:**
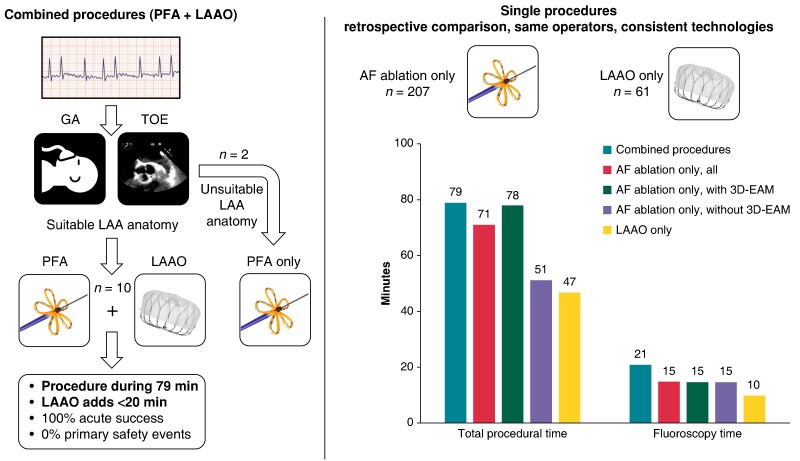

**What’s new?**

- Simultaneous left atrial appendage (LAA) occlusion and atrial fibrillation ablation utilizing pulsed field ablation is feasible and safe.
- In patients with a suitable LAA anatomy, the combined procedure can be executed within a short overall procedure duration.
- Left atrial appendage occlusion adds <20 min to the overall procedure duration.

## Introduction

Catheter ablation is the preferred treatment for patients experiencing symptomatic atrial fibrillation (AF). Pulsed field ablation (PFA), a novel ablation technology, demonstrates enhanced efficacy and safety compared with traditional, purely thermal ablation methods.^1,2^ In the absence of conclusive data, patients at high thromboembolic risk typically continue oral anticoagulation therapy even after successful AF ablation. Left atrial appendage occlusion (LAAO) offers an alternative to oral anticoagulation for patients with AF, especially those with a history of bleeding, or those who are unwilling or unable to maintain oral anticoagulation treatment.^3^ With detailed, pre-procedural imaging of the left atrial appendage (LAA) and peri-interventional guiding by transoesophageal echocardiography (TOE), LAAO has evolved into a procedure that is both safe and efficient.^4–6^ While pulmonary vein isolation (PVI) and LAAO are both conducted in the left atrium and share the same access route, their combination is not conventionally practiced. However, technological advancements that enhance the safety and efficiency of PVI through PFA and reduce procedure duration, along with improvements in the design and implementation process of LAA occluders, make the concurrent application of these procedures more attractive. Furthermore, combining both interventions into a single procedure, rather than staging them in two separate procedures, provides an opportunity to reduce overall healthcare costs. In this report, we detail our initial experience with the simultaneous performance of PVI using PFA and LAAO in patients with favourable LAA anatomy.

## Methods

Since August 2021, we have initiated a programme of AF ablation using PFA and concomitant LAAO for patients who are candidates for both treatments and have favourable LAA anatomies. For this combined procedure, we exclusively utilized the Watchman FLX occluder (Boston Scientific, MA, USA) for LAAO. Patients presenting with a complex LAA anatomy, as determined by intra-procedural imaging or pre-procedural cardiac computed tomography angiography (CCTA), were excluded from our study. This report details the outcomes of the initial patients who underwent the combined procedure of AF ablation and LAAO until the end of 2023, along with the characteristics of those who were excluded. The combined safety endpoint included death, cerebrovascular event, systemic embolism, major bleeding (BARC 3–5), clinically relevant pericardial effusion, device embolization, or acute kidney injury occurring within 7 days after LAAO. All individuals receiving AF ablation or LAAO at our institution are prospectively included in dedicated AF ablation and LAAO registries. Participation in the registries requires the provision of written informed consent from each patient. Both registries have received approval from the local ethics committee and are conducted in accordance with the Declaration of Helsinki. To compare procedural characteristics, such as procedure duration, fluoroscopy time, and radiation dose, between combined procedures (AF ablation and LAAO) and single procedures (either AF ablation alone or LAAO alone), we aggregated and analysed data from the two registries, including procedures performed during the same time period by the same team of operators. For AF ablation, we included procedures conducted using only PFA. For LAAO, only those employing the Watchman FLX LAA occluder were considered.

### Concomitant atrial fibrillation ablation and left atrial appendage occlusion procedures

**Figure 1:**
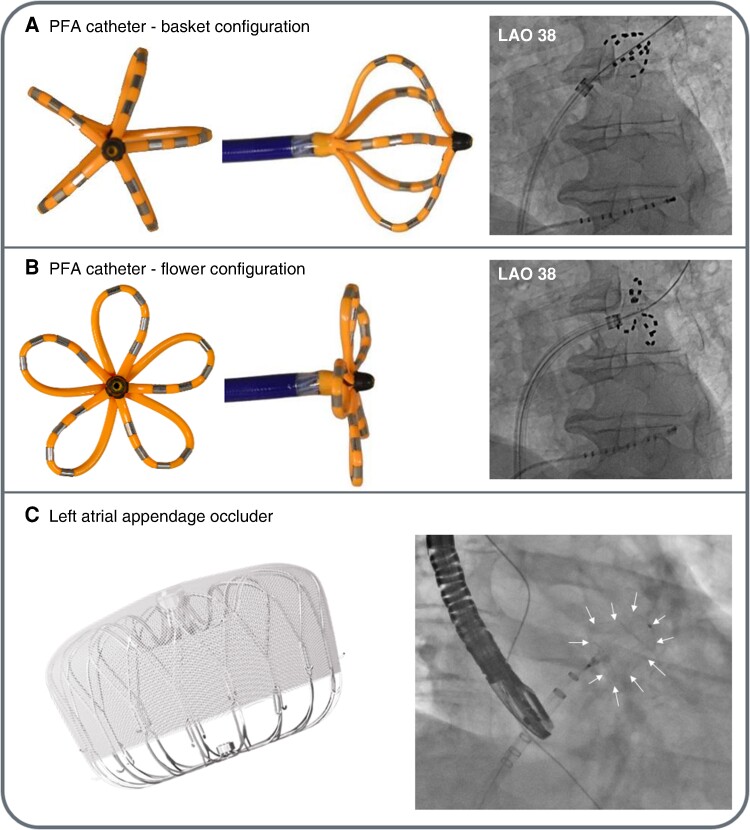
An over-the-wire, 31 mm multi-polar pulsed field ablation catheter. (*A*) The front view, side view, and X-ray image with the catheter located in the left superior pulmonary vein (LSPV) ostium, all in the basket configuration. (*B*) Further deployment of the catheter results in the ‘flower’ configuration. All electrodes are active during ablation, and rotation of the device between applications ensures complete circumferential pulmonary vein isolation. (*C*) A view of the left atrial appendage occluder and X-ray image of the occluder implanted in the left atrial appendage. LAO, left anterior oblique view; PFA, pulsed field ablation.

Patients scheduled for concomitant PVI and LAAO underwent a CCTA scan prior to the procedure to rule out left atrial thrombus and to assess LAA anatomy. The combined procedure was performed under general anaesthesia. Venous access was obtained with ultrasound guidance. Transseptal puncture with a Brockenbrough needle was aimed at an inferior, posterior site, guided by TOE imaging, either directly using the Faradrive sheath (Boston Scientific) or a standard transseptal sheath that was then exchanged over the wire for the Faradrive sheath.^7^ Heparin was administered after obtaining left atrial access to achieve an activated clotting time target of >350 s. A three-dimensional (3D) electroanatomic mapping system was used in the first three cases and in both redo cases. For PVI or posterior wall ablation (PWA), the Farawave catheter and the Farastar generator (Boston Scientific) were used, as described elsewhere.^8,9^ For PVI, a minimum of four applications at 2 kV were delivered in both the basket and the flower configurations to each of the pulmonary veins (*Figure* *1A* and *B*). For PWA, four anchor lesions to each vein were delivered with the catheter in flower configuration, wired pulmonary vein, and with posterior torque to the sheath and catheter. Additional PFA applications in flower configuration and with retracted wire were applied to cover the entire posterior wall.^9^ Successful, acute PVI and PWA were verified by 3D electroanatomic mapping or, for PVI, by using the Farawave catheter in a basket configuration in all pulmonary veins for the assessment of entrance and exit block.^8^

Once ablation was completed, the Farawave sheath was exchanged over a stiff 0.035″ wire for the Watchman delivery sheath (Boston Scientific). Left atrial appendage angiography, coupled with precise measurements of the LAA via TOE, was employed to accurately size the Watchman FLX device (Boston Scientific). Subsequently, LAAO was executed in adherence to the prescribed instructions for use and in alignment with the latest guidelines (*Figure 1C*).

### Follow-up

All patients were followed up after 3, 6, and 12 months post-ablation to screen for atrial arrhythmias, usually with a 7-day Holter electrocardiogram (ECG), supplemented by a 12-lead ECG in the event of symptomatic episodes. Recurrence of atrial arrhythmia was defined as episodes of AF, flutter, or tachycardia lasting longer than 30 s. To assess the positioning of the left LAAO, detect peri-device leaks, and identify any device-related thrombi, all patients received a TOE and/or a CCTA 45 days after the procedure.

### Statistical analysis

Continuous variables, when not normally distributed, are reported as medians with their respective ranges. Categorical variables are expressed in terms of frequencies and percentages.

## Results

### Patient characteristics

**Figure 2:**
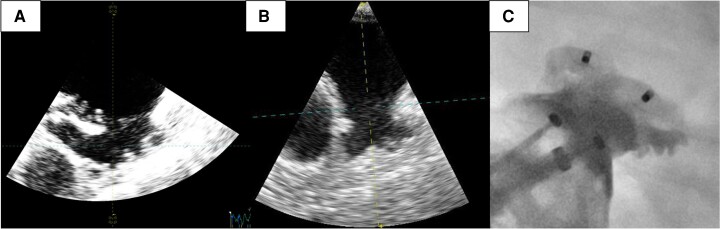
A left atrial appendage anatomy of excluded cases. (*A*) A left atrial appendage with a chicken wing configuration and insufficient depth for the placement of a Watchman FLX LAAO. (*B*) A left atrial appendage with a bi-lobulated configuration. (*C*) Closure of the bi-lobulated left atrial appendage employing two 16 mm Amplatzer occluders. LAAO, left atrial appendage occlusion.

Between August 2021 and December 2023, we successfully performed concomitant AF ablation using PFA and LAAO in 10 patients. Details of patient demographics are presented in *Table 1*. Among these, eight patients underwent first AF ablation procedures and the remaining two had redo procedures. The indications for LAAO included a history of bleeding in seven patients (70%), personal preference in two (20%), and intolerance to oral anticoagulants in one (10%). Two candidates were not considered for the combined treatment due to complex LAA anatomies, as depicted in *Figure 2*. One patient, with a history of gastrointestinal bleeding, underwent LAAO 3 months later, employing two 16 mm Amplatzer occluders to seal a bi-lobulated LAA configuration. The second patient opted out of the initially scheduled LAAO procedure, based on personal preference.

**Table 1:** Baseline characteristics of patients undergoing concomitant atrial fibrillation ablation and left atrial appendage occlusion

Patient characteristics	*n* = 10
Age, years	70 (62–76)
Male sex	5 (50%)
Hypertension	7 (70%)
Diabetes mellitus	1 (10%)
History of ischaemic stroke or transient ischaemic attack	—
Coronary artery disease	5 (50%)
History of arterial embolism	1 (10%)
**CHAD_2_DS_2_VASc** score	2.5 (2–4)
HAS-BLED score	3 (1–4)
Paroxysmal AF	4 (40%)
Persistent AF	6 (60%)
Left ventricular ejection fraction, %	65 (40–65)
Bleeding history	7 (70%)
Intracranial bleeding history	1 (10%)
Gastrointestinal bleeding history	3 (30%)

The values shown are numbers with percentages in parentheses or median with range.

### Procedural characteristics

**Figure 3:**
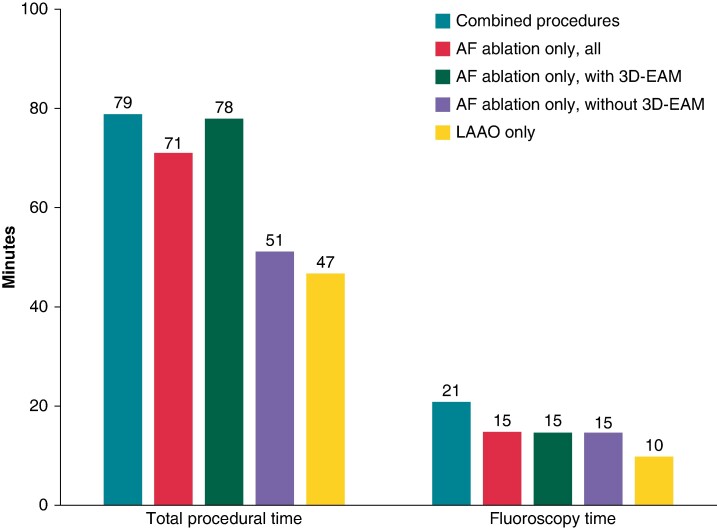
Bar graphs showing the respective procedure and fluoroscopy times for the combined and single procedures. 3D-EAM, three-dimensional electroanatomic mapping; AF, atrial fibrillation; LAAO, left atrial appendage occlusion.

In the eight patients with first AF ablation, only PVI was performed. In the two redo procedures, reconnected veins were re-isolated and the posterior wall was targeted for ablation. A 3D electroanatomic mapping system was used in both redo procedures and in three first AF ablation procedures. The median size of the implanted Watchman FLX device was 24 mm (range 20–27 mm). For single-procedure comparison, we aggregated 207 AF ablation procedures conducted using PFA and 61 LAAO procedures using the Watchman FLX LAA occluder. Among the 207 AF ablation procedures, 137 patients (66%) had paroxysmal AF and 139 (67%) had first AF ablation procedures. A 3D electroanatomic mapping system was employed in 152 of the AF ablation cases (73%). Among the LAAO procedures, the median size of the Watchman FLX LAA occluder was 24 mm (range 20–35 mm). The procedural characteristics of the combined and single procedures are shown in *Table 2* and *Figure 3*. The median procedure time was 79 min (range 60–125 min) for the combined procedure, compared with 71 min for individual PFA-based AF ablation (25–241 min) and 47 min for LAAO (15–162 min). Individual PFA-based AF ablation was 51 min without and 78 min with 3D electroanatomic mapping during the procedure. Overall, LAAO added 20 min (15–37 min) to overall procedure length in the combined procedure. The fluoroscopy time of the combined procedure increased by a median of 6 and 11 min compared with individual PFA-based AF ablation and LAAO, respectively (*Table 2* and *Figure 3*).

**Table 2:** Procedural characteristics of patients undergoing concomitant or single procedures

Procedural characteristics	Combined procedures *n* = 10	AF ablation only *n* = 207			LAAO only *n* = 61
		All	With 3D-EAM *n* = 152	Without 3D-EAM *n* = 55	
Total procedural time, min	79 (60–125)	71 (25–241)	78 (37–241)	51 (25–189)	47 (15–162)
** **Vein access to transseptal puncture, min	10 (2–27)	—	—	—	—
** **First to last PFA application, min	24 (17–43)	—	—	—	—
** **Transseptal puncture to last PFA application, min	38 (27–79)	—	—	—	—
** **Vein access to last PFA application, min	49 (34–93)	—	—	—	—
** **Last PFA application to occluder insertion, min	14 (10, 29)	—	—	—	—
** **LAA occluder insertion to release, min	5 (2–17)	—	—	—	—
** **Last PFA application to LAA occluder release, min	20 (15–37)	—	—	—	—
Fluoroscopy time, min	21 (15–26)	15 (5–44)	15 (5–44)	15 (6–30)	10 (3–50)
Radiation dose, cGy cm^2^	1539 (191–10 323)	431 (52–46 527)	445 (73–46 527)	367 (52–10 323)	2476 (874–32 721)

The values shown are numbers with percentages in parentheses or medians with ranges.

3D-EAM, three-dimensional electroanatomic mapping; LAA, left atrial appendage; LAAO, left atrial appendage occlusion; PFA, pulsed field ablation.

### Procedural safety

No safety endpoint occurred. One patient experienced minor haemoptysis on the day following the procedure, which resolved without intervention. No further adverse events were reported.

### Follow-up

Post-procedure, oral anticoagulation therapy was maintained for 3 months across all patients, after which it was discontinued. Thereafter, either acetylsalicylic acid or clopidogrel was prescribed for 12 months in five patients (50%) and indefinitely in the remaining five patients (50%) due to coronary artery disease. Transoesophageal echocardiography and/or CCTA 45 days after the procedure were performed in all patients (eight patients had both TOE and CCTA, one patient had only TOE, and one had only CCTA), which showed no device-related thrombus and the correct position of the LAAO. Five patients showed in CCTA LAA patency without intra- or peri-device leaks, whereas two patients showed intra-device leaks, one showed a mixed intra- or peri-device leak, and one showed neither a leak nor patency. After a mean follow-up of 9 months (range 3–12 months), three patients (30%) had arrhythmia recurrence.

## Discussion

Our initial experience in 10 patients indicates that concomitant PFA-based AF ablation and LAAO can be conducted safely and with high procedural efficacy. The median procedure duration was only 79 min, and the added LAAO increased the procedure duration by only 20 min beyond the last PFA application. Conversely, at our centre, the duration of PFA-based AF ablation with and without the use of 3D electroanatomic mapping was 78 and 51 min, respectively, while the time for LAAO performed independently was 47 min. Similarly, the fluoroscopy time for the combined procedure exhibited a modest increase to 21 min, extending only by 6 min compared with PFA-based AF ablation alone, and by 11 min when compared with LAAO performed singly.

In comparison, the largest published multi-centre, prospective registry, which includes data on combined LAAO and AF ablation procedures from the pre-PFA era, reported a mean procedural time of 177 min and a mean fluoroscopy time of 31 min. Radiofrequency ablation was employed in the majority of these cases.^10^ Another study, which utilized cryoablation for AF ablation, reported a mean procedure time of 148 min, with cryoablation accounting for an average of 107 min and LAAO requiring 40 min.^11^ Pulsed field ablation–based AF ablation is recognized for its shorter procedure duration compared with AF ablation involving thermal energies.^12^ Indeed, in the hands of experienced operators, the mean procedure duration of PFA-based AF ablation is typically <1 h.^13^ Additionally, the duration of LAAO can be further minimized through the pre-selection of patients with anatomies suitable for the procedure. This strategic combination has enabled the further reduction in procedure duration relative to those reported in published studies. Importantly, this also contributes to decreased left atrial dwell time, which is relevant, as prolonged left atrial dwell times are linked to increased procedural risks.^14^ A pre-procedural CCTA scan is not mandatory, as the procedure can be effectively performed using intra-procedural TOE alone. However, the advantage of a pre-procedural CCTA scan lies in its ability to clarify the LAA anatomy, which can aid in procedural planning. For instance, in cases of complex anatomy, it may be preferable to conduct two separate procedures instead of a concomitant one.

Radiofrequency ablation can result in considerable oedema formation, which increases the thickness of the left atrial ridge following the isolation of the left superior pulmonary vein.^15^ In contrast, PFA is associated with less oedema formation.^16^ This reduced oedema formation is another advantage of PFA over thermal energies, as it has the potential to improve LAAO outcomes by facilitating more accurate device sizing and placement, thereby enhancing the overall safety and efficacy of the combined procedure.

Strategically, our approach for the combined procedure begins with AF ablation and concludes with LAAO for two primary reasons. First, this sequence minimizes the risk of occluder displacement during the manipulation of the left pulmonary veins. Secondly, it avoids the potential for PFA energy short-circuiting during the ablation of the left superior pulmonary vein. This concern is particularly relevant with the Amplatzer Amulet LAA occluder, because its disc that protrudes out of the LAA may come into contact with the electrodes on the splines of the PFA catheter during isolation of the left superior pulmonary vein.^17^

Previous analyses from multi-centre registries have reported low rates of adverse events associated with the concurrent performance of AF ablation and LAAO.^18,19^ Similarly, the long-term efficacy of the combined procedure, in terms of both AF-free survival and the prevention of embolic events, has yielded favourable results.^20^ To date, only few cases have been published of PFA-based AF ablation and concomitant LAAO.^21,22^ As the number of PFA procedures continues to increase rapidly, it is likely that we will soon see larger studies emerge that report on the concurrent use of PFA-based AF ablation and LAAO. These studies will enhance our understanding of the safety and efficacy of this combined approach.

Two pivotal randomized controlled trials are currently underway, which may further pave the way for the combination of these procedures. First, the OCEAN trial is designed to assess the need for continued oral anticoagulation following successful AF ablation.^23^ Secondly, the OPTION trial aims to determine whether LAAO is non-inferior to oral anticoagulation in patients after PVI.^24^ These trials may significantly influence clinical practice by providing evidence-based guidance on the management of patients’ post-AF ablation and boost the number of combined procedures in the future. In the light of this, the combination of PFA-based AF ablation with LAAO may come in very handy.

### Limitations

As a retrospective analysis, this study is subject to the inherent limitations characteristic of its design. Notably, our cohort of patients undergoing the combined procedure is small, and larger studies are necessary to verify these preliminary findings.

## Conclusions

Simultaneous PFA-based AF ablation and LAAO using the Watchman FLX device in patients with a suitable LAA anatomy is feasible and safe and can be performed within a short overall procedure duration. Adding LAAO to PFA-based AF ablation prolongs the procedure time by <20 min.

## Data Availability

All relevant data are within the manuscript.
